# Supplementary material for: First detection and characterization of mcr-1 colistin resistant E. coli from wild rat in Bangladesh
Source: PLoS One. 2024 May 14;19(5):e0296109. doi: 10.1371/journal.pone.0296109 (PMC11093362; doi:10.1371/journal.pone.0296109)
Supplement: S1 Fig — A) Distribution of CDS, ARGs, CRISPR-Cas systems and prophages. The circular image shows the GC skew (Ring 1 from inside), GC content (Ring 2), CDS derived through Prokka annotation (Ring 3), ARGs from CARD analysis (Ring 4), CRISPR/Cas clusters (Ring 5) and prophages derived by Phigaro tools (Ring 6). B) Linear view of the Cas-cluster in the RJWEc-MCR-1-BAU genome. Circular view of the genome and other systems were prepared using Proksee tools (Prokka, CARD Resistance Gene Identifier, CRISPR/Cas Finder and Phigaro (https://proksee.ca/). (PPTX) [file pone.0296109.s001.pptx]

## Slide 1
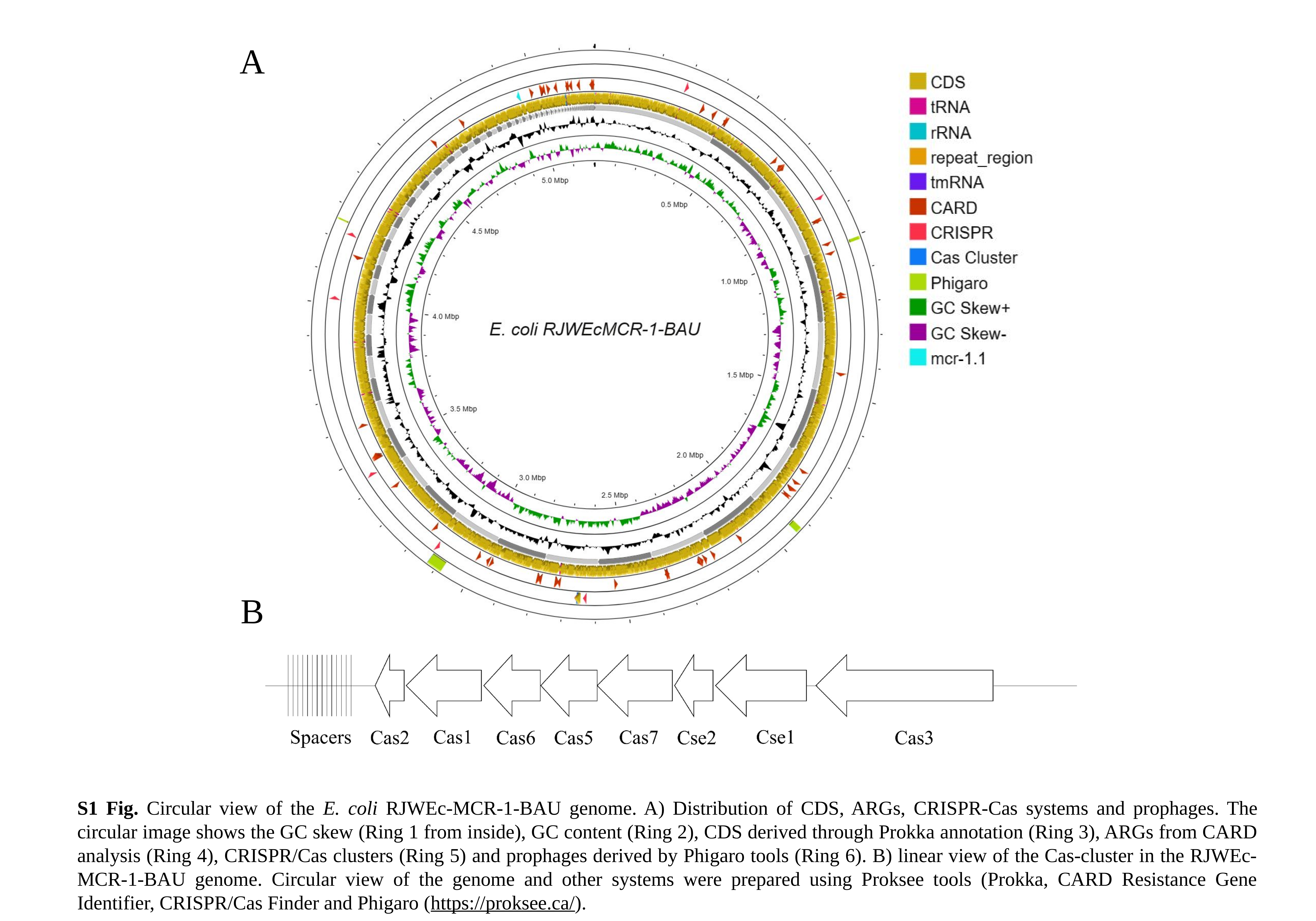

A
B
S1 Fig. Circular view of the E. coli RJWEc-MCR-1-BAU genome. A) Distribution of CDS, ARGs, CRISPR-Cas systems and prophages. The circular image shows the GC skew (Ring 1 from inside), GC content (Ring 2), CDS derived through Prokka annotation (Ring 3), ARGs from CARD analysis (Ring 4), CRISPR/Cas clusters (Ring 5) and prophages derived by Phigaro tools (Ring 6). B) linear view of the Cas-cluster in the RJWEc-MCR-1-BAU genome. Circular view of the genome and other systems were prepared using Proksee tools (Prokka, CARD Resistance Gene Identifier, CRISPR/Cas Finder and Phigaro (https://proksee.ca/).
