# Supplementary material for: First detection and characterization of mcr-1 colistin resistant E. coli from wild rat in Bangladesh
Source: PLoS One. 2024 May 14;19(5):e0296109. doi: 10.1371/journal.pone.0296109 (PMC11093362; doi:10.1371/journal.pone.0296109)
Supplement: S2 Fig — (PPTX) [file pone.0296109.s002.pptx]

## Slide 1
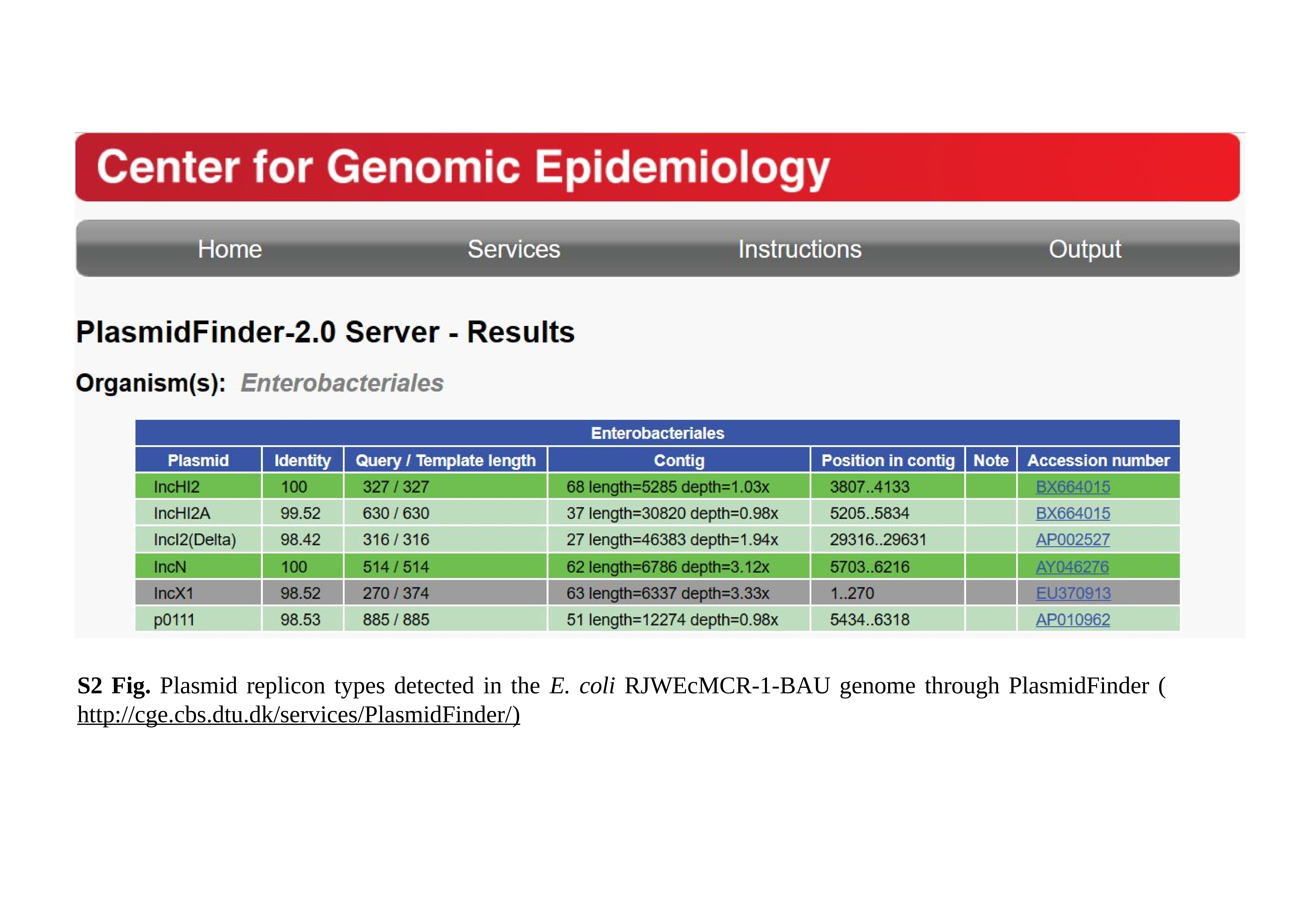

S2 Fig. Plasmid replicon types detected in the E. coli RJWEcMCR-1-BAU genome through PlasmidFinder (http://cge.cbs.dtu.dk/services/PlasmidFinder/)
