# Supplementary material for: First detection and characterization of mcr-1 colistin resistant E. coli from wild rat in Bangladesh
Source: PLoS One. 2024 May 14;19(5):e0296109. doi: 10.1371/journal.pone.0296109 (PMC11093362; doi:10.1371/journal.pone.0296109)
Supplement: S3 Fig — The evolutionary history was inferred using the Neighbor-Joining method [1]. The optimal tree is shown. The tree is drawn to scale, with branch lengths in the same units as those of the evolutionary distances used to infer the phylogenetic tree. The evolutionary distances were computed using the p-distance method [2] and are in the units of the number of base differences per site. This analysis involved 35 nucleotide sequences. All ambiguous positions were removed for each sequence pair (pairwise deletion option). There were a total of 1674 positions in the final dataset. Evolutionary analyses were conducted in MEGA11 [3]. The strain described in this study is enclosed with red rectangle. (PPTX) [file pone.0296109.s003.pptx]

## Slide 1
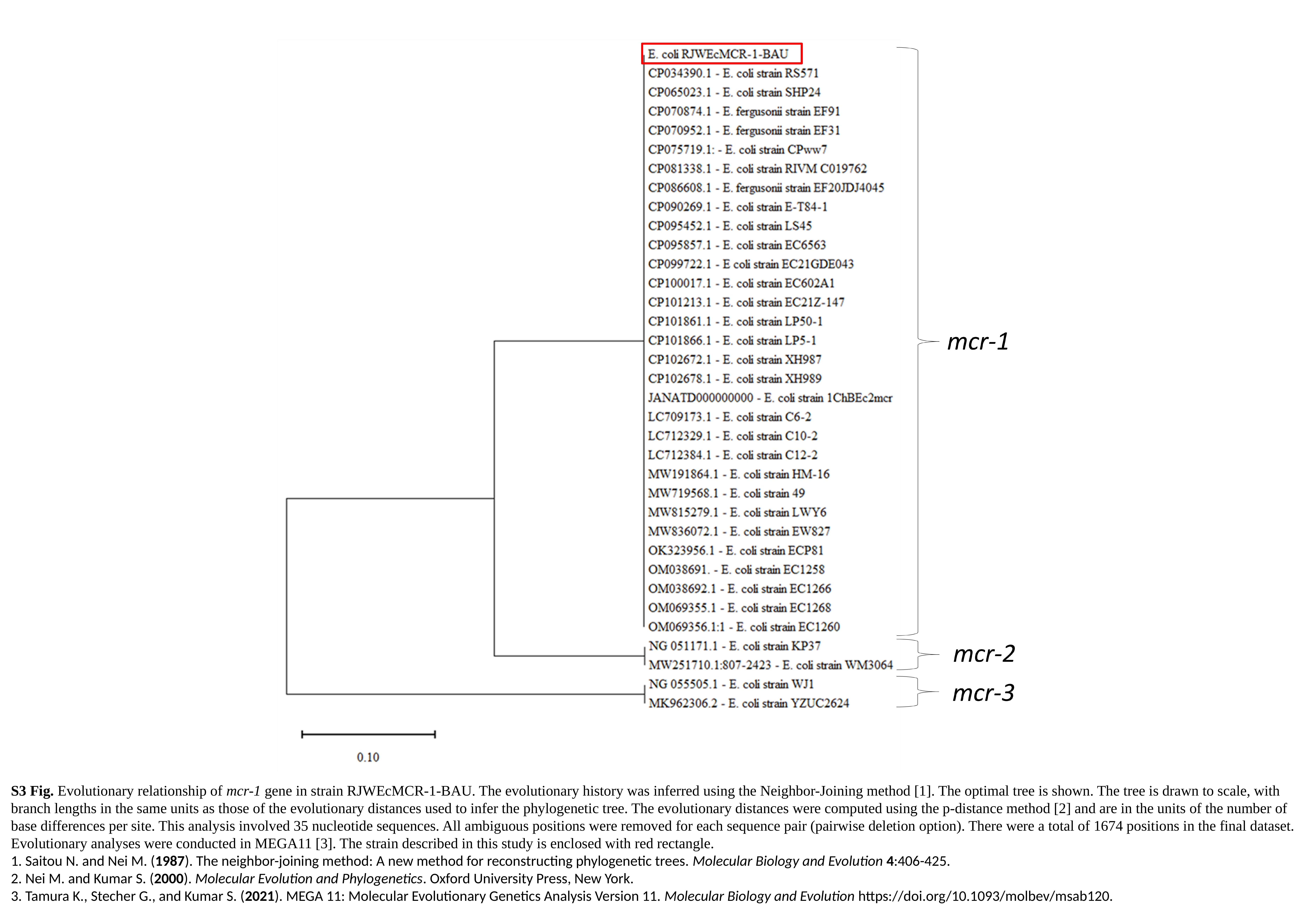

S3 Fig. Evolutionary relationship of mcr-1 gene in strain RJWEcMCR-1-BAU. The evolutionary history was inferred using the Neighbor-Joining method [1]. The optimal tree is shown. The tree is drawn to scale, with branch lengths in the same units as those of the evolutionary distances used to infer the phylogenetic tree. The evolutionary distances were computed using the p-distance method [2] and are in the units of the number of base differences per site. This analysis involved 35 nucleotide sequences. All ambiguous positions were removed for each sequence pair (pairwise deletion option). There were a total of 1674 positions in the final dataset. Evolutionary analyses were conducted in MEGA11 [3]. The strain described in this study is enclosed with red rectangle.
1. Saitou N. and Nei M. (1987). The neighbor-joining method: A new method for reconstructing phylogenetic trees. Molecular Biology and Evolution 4:406-425.
2. Nei M. and Kumar S. (2000). Molecular Evolution and Phylogenetics. Oxford University Press, New York.
3. Tamura K., Stecher G., and Kumar S. (2021). MEGA 11: Molecular Evolutionary Genetics Analysis Version 11. Molecular Biology and Evolution https://doi.org/10.1093/molbev/msab120.
