# Supplementary material for: First detection and characterization of mcr-1 colistin resistant E. coli from wild rat in Bangladesh
Source: PLoS One. 2024 May 14;19(5):e0296109. doi: 10.1371/journal.pone.0296109 (PMC11093362; doi:10.1371/journal.pone.0296109)
Supplement: S1 Table — (DOCX) [file pone.0296109.s004.docx]

**S1 Table: Primers used in this study**

| **Purpose** | **Primer Name** | **Sequence (5’→3’)** | **Target Gene** | **Product Size (bp)** | **PCR conditions** | | | | **Reference** |
| --- | --- | --- | --- | --- | --- | --- | --- | --- | --- |
|  |  |  |  |  | **Denaturation** | **Annnealing** | **Extension** | **Cycles** |  |
| Identification of *E. coli* | ECO-1 | GACCTCGGTTTAGTTCACAGA | *mal B* | 585 | 94^o^C, 45 s | 58 ^o^C, 45 s | 72 ^o^C, 60 s | 30 | (Wang et al., 1996) |
|  | ECO-2 | CACACGCTGACGCTGACCA |  |  |  |  |  |  |  |
| MCR detection | *mcr1*_320bp_fw | AGTCCGTTTGTTCTTGTGGC | *mcr-1* | 320 | 94^o^C, 30 s | 58 ^o^C, 90 s | 72 ^o^C, 60 s | 25 | (Rebelo et al., 2018) |
|  | *mcr1*_320bp_rev | AGATCCTTGGTCTCGGCTTG |  |  |  |  |  |  |  |
|  | *mcr2*_700bp_fw | CAAGTGTGTTGGTCGCAGTT | *mcr-2* | 715 |  |  |  |  |  |
|  | *mcr2*_700bp_rev | TCTAGCCCGACAAGCATACC |  |  |  |  |  |  |  |
|  | *mcr3*_900bp_fw | AAATAAAAATTGTTCCGCTTATG | *mcr-3* | 929 |  |  |  |  |  |
|  | *mcr3*_900bp_rev | AATGGAGATCCCCGTTTTT |  |  |  |  |  |  |  |
|  | *mcr4*_1100bp_fw | TCACTTTCATCACTGCGTTG | *mcr-4* | 1,116 |  |  |  |  |  |
|  | *mcr4*_1100bp_rev | TTGGTCCATGACTACCAATG |  |  |  |  |  |  |  |
|  | *MCR5*_fw | ATGCGGTTGTCTGCATTTATC | *mcr-5* | 1,644 |  |  |  |  |  |
|  | *MCR5*_rev | TCATTGTGGTTGTCCTTTTCTG |  |  |  |  |  |  |  |
|  | *mcr-9-mp-fw* | TTCCCTTTGTTCTGGTTG | *mcr-9* | 1011 | 95^o^C, 30 s | 55^o^C 30 s | 72^o^C, 60 s | 30 | Göpel et al., 2024 |
|  | *mcr-9-mp-rev* | GCAGGTAATAAGTCGGTC |  |  |  |  |  |  |  |
|  | *mcr-10-mp-F* | TATCCTGAGCCGTCTTGAAC | *mcr-10* | 386 |  |  |  |  |  |
|  | *mcr-10-mp-R* | GGATCAGCGAAGCGAGCAT |  |  |  |  |  |  |  |
| Phylogenetic Grouping | ChuA.1 | GACGAACCAACGGTCAGGAT | *chuA* | 279 | 94^o^C, 30 s | 55^o^C 30 s | 72^o^C, 30 s | 30 | (Clermont et al., 2000) |
|  | ChuA.2 | TGCCGCCAGTACC AAAGACA |  |  |  |  |  |  |  |
|  | YjaA.1 | TGAAGTGTCAGGAGACGCTG | *yjaA* | 211 |  |  |  |  |  |
|  | YjaA.2 | ATGGAGAATGCGTTCCTCAAC |  |  |  |  |  |  |  |
|  | TspE4C2.1 | GAGTAATGTCGGGGCATTCA | TSPE4.C2 | 152 |  |  |  |  |  |
|  | TspE4C2.2 | CGCGCCAACAAAGTATTACG |  |  |  |  |  |  |  |
|  |  |  |  |  |  |  |  |  |  |
| Wang RF, Cao WW, Cerniglia CE. PCR detection and quantitation of predominant anaerobic bacteria in human and animal fecal samples. Appl Environ Microbiol 1996;62:1242–7. https://doi.org/10.1128/AEM.62.4.1242-1247.1996. | | | | | | | | | |
| Rebelo AR, Bortolaia V, Kjeldgaard JS, Pedersen SK, Leekitcharoenphon P, Hansen IM, et al. Multiplex PCR for detection of plasmid-mediated colistin resistance determinants, mcr-1, mcr-2, mcr-3, mcr-4 and mcr-5 for surveillance purposes. Euro Surveill 2018;23. https://doi.org/10.2807/1560-7917.ES.2018.23.6.17-00672. | | | | | | | | | |
| Clermont O, Bonacorsi S, Bingen E. Rapid and simple determination of the Escherichia coli phylogenetic group. Appl Environ Microbiol 2000;66:4555–8. https://doi.org/10.1128/AEM.66.10.4555-4558.2000. | | | | | | | | |  |
