# Supplementary material for: First detection and characterization of mcr-1 colistin resistant E. coli from wild rat in Bangladesh
Source: PLoS One. 2024 May 14;19(5):e0296109. doi: 10.1371/journal.pone.0296109 (PMC11093362; doi:10.1371/journal.pone.0296109)
Supplement: S2 Table — (DOCX) [file pone.0296109.s005.docx]

**S2 Table. Antimicrobial agents used in this study, along with their disc concentration, and classes**

| **Antibiotics** | **Disc concentration** | **Antibiotic class** |
| --- | --- | --- |
| Amoxicillin (AML) | 10 µg | Penicillins |
| Ampicillin (AMP) | 10 µg |  |
| Ceftriaxone (CTR) | 30 µg | Cephalosporins |
| Cefuroxime (CXM) | 30 µg |  |
| Ciprofloxacin- (CIP) | 5 µg | Quinolones |
| Doxycycline (DO) | 30 µg | Tetracyclines |
| Azithromycin (AZM) | 15 µg | Macrolides |
| Erythromycin (E) | 15 µg |  |
| Enrofloxacin (ENR) | 5 µg | Quinolones |
| Fosfomycin (FOS) | 50 µg | Phosphonic |
| Gentamycin (CN) | 10 µg | Aminoglycosides |
| Levofloxacin (LEV) | 5 µg | Quinolones |
| Imipenem (IMP) | 10 µg | Carbapenems |
| Meropenem (MEM) | 10 µg |  |
| Moxifloxacin (MXF) | 5 µg | Quinolones |
| Nalidixic acid (NA) | 30 µg |  |
| Nitrofurantoin (F) | 300 µg | Nitrofuran |
| Neomycin (N) | 30 µg | Aminoglycosides |
| Streptomycin (S) | 10 µg |  |
| Sulfamethoxazole-Trimethoprim (SXT) | 25 µg | Sulfonamides |
| Tetracycline (TE) | 30 µg | Tetracyclines |
