# Supplementary material for: First detection and characterization of mcr-1 colistin resistant E. coli from wild rat in Bangladesh
Source: PLoS One. 2024 May 14;19(5):e0296109. doi: 10.1371/journal.pone.0296109 (PMC11093362; doi:10.1371/journal.pone.0296109)
Supplement: S3 Table — (DOCX) [file pone.0296109.s006.docx]

**S3 Table. *E. coli* Plasmid sequences used as a reference in this study**

| **Plasmid names** | **Accession no.** | **Sources** |
| --- | --- | --- |
| pHLJ111-101 | MN232204.1 | Chicken |
| pHLJ111-5 | MN232208.1 | Chicken |
| pHLJ179-167 | MN232211.1 | KY657478.1 |
| pMCR-H9 | NZ_CP029184.1 | Homo sapiens |
| pUSU-ECO-12704_4 | KY657478.1 | Homo sapiens |
| pAH81-113 | MN232185.1 | Chicken |
| pHLJ109-25 | MN232198.1 | Chicken |
| pZJ3920-3 | NZ_CP020548.1 | Homo sapiens |
| pEc_20COE13 | KY012274.1 | Homo sapiens |
| pSH13G1582 | MH522412.1 | Homo sapiens |
| pLWY24J-mcr-1.1 | MN689940.1 | chicken |
| pEC5-1 | CP016185.1 | - |
| pColR644SK1 | MF175188.1 | Homo sapiens |
| pColR598_2 | MF175189.1 | Homo sapiens |
| pPK411 | MK571810.1 | Chicken |
| pHAB-6 | MK574668.1 | - |
| pHLJ111-20 | MN232206.1 | Chicken |
| pSDC-F2_12BHI2 | MH287085.1 | Pig |
| p280_40A | CP031284.1 | Chicken |
| pRHB30-C20_2 | CP055946.1 | Pig |
| pRHB28_C21_2 | CP057358.1 | Pig |
| pYD786-1 | KU254578.1 | Homo sapiens |
| pK18EC051 | CP049300.1 | Pig |
