# Supplementary material for: First detection and characterization of mcr-1 colistin resistant E. coli from wild rat in Bangladesh
Source: PLoS One. 2024 May 14;19(5):e0296109. doi: 10.1371/journal.pone.0296109 (PMC11093362; doi:10.1371/journal.pone.0296109)
Supplement: S3 Data — (DOCX) [file pone.0296109.s009.docx]

**Analysis of chromosomal genes of RJWEcMCR-1-BAU for mutations conferring colistin resistance**

Amino acid sequences in **bold** indicates **not specified mutation**, while **bold and underlined** letters indicate **specified mutation** according to Binsker et al., 2022.

> PmrA

MKILIVEDDTLLLQGLILAAQTEGYACDGVTTARMAEQSLEAGHYSLVVLDLGLPDEDGLHFLARIRQKKYTLPVLILTARDTLTDKIAGLDVGADDYLVKPFALEELHARIRALLQRHNNQGESELIVGNLTLNMGRRQVWMGGEELILTPKEYALLSRLMLKAGSPVHREILYNDIYNWDNEPSTNTLEVHIHNLRDKVGKARIRTVRGFGYMLVANEEN

> PmrB

MHFLRRPISLRQRLILTIGAILLVFELISVFWLWHESTEQIQLFEQALRDNRNNDRHIMREIREAVASLIVPGVFMVSLTLFICYQAVRRITRPLAELQKELEARTADNLTPIAIHSATLEIEAVVSALNDLVSRLTSTLDNERLFTADVAHELRTPLAGVRLHLELLAKTHHIDVAPLVARLDQMMESVSQLLQLARAGQSFSSGNYQHVKLLEDVILPSYDELSTMLDQRQQTLLLPESAADITVQGDATLLRMLLRNLVENAHRYSPQGSNIMIKLQED**G**GAVMAVEDEGPGIDESKCGELSKAFVRMDSRYGGIGLGLSIVSRITQLHHGQFFLQNRQETSGTRAWVRLKKDQ**N**VANQI

> PmrC or EptA

MLKRLLKRPSLNLLAWLLLAAFYISICLNIAFFKQVLQALPLDSLHNVLVFLSMPVVAFSVINIVLTLSSFLWLNRPLACLFILVGAAAQYFIMTYGIVIDRSMIANIIDTTPAESYALMTPQMLLTLGFSGVLAALIACWIKIKPATSRLRSVLFRGANILVSVLLILLVAALFYKDYASLFRNNKELVKSLSPSNSIVASWSWYSHQRLANLPLVRIGEDAHRNPLMQNEKRKNLTILIVGETSRAENFSLNGYPRETNPRLAKDNVVYFPNTASCGTATAVSVPCMFSDMPREHYKEELAQHQEGVLDIIQRAGINVLWNDNDGGCKGACDRVPHQNVTALNLPGQCINGECYDEVLFHGLEEYINNLQGDGVIVLHTIGSHGPTYYNRYPPQFRKFTPTCDTNEIQTCSKEQLVNTYDNTLVYVDYIVDKAINLLKEHQDKFTTSLVYLSDHGESLGENGIYLHGLPYAIAPDSQKQVPMLLWLSEDYQKRYQVDQNCLQKQAQTQHYSQDNLFSTLLGLTGVETKYYQAADDILQTCRRVSE

> PhoP MRVLVVEDNALLRHHLKVQIQDAGHQVDDAEDAKEADYYLNEHLPDIAIVDLGLPDEDGLSLIRRWRSNDVSLPILVLTARESWQDKVEVLSAGADDYVTKPFHIEEVMARMQALMRRNSGLASQVISLPPFQVDLSRRELSINDEVIKLTAFEYTIMETLIRNNGKVVSKDSLMLQLYPDAELRESHTIDVLMGRLRKKIQAQYPQEVITTVRGQGYLFELR

> PhoQ

MKKLLRLFFPLSLRVRFLLATAAVVLVLSLAYGMVALIGYSVSFDKTTFRLLRGESNLFYTLAKWENNKLHVELPENIDKQSPTMTLIYDENGQLLWAQRDVPWLMKMIQPDWLKSNGFHEIEADVNDTSLLLSGDHSIQQQLQEVREDDDDAEMTHSVAVNVYPATSRMPKLTIVVVDTIPVELKSSYMVWSWFIYVLSANLLLVIPLLWVAAWWSLRPIEALAKEVRELEEHNRELLNPATTRELTSLVRNLNRLLKSERERYDKYRTTLTDLTHSLKTPLAVLQSTLRSLRSEKMSVSDAEPVMLEQISRISQQIGYYLHRASMRGGTLLSRELHPVAPLLDNLTSALNKVYQRKGVNISLDISPEISFVGEQNDFVEVMGNVLDNACKYCLEFVEISARQTDEHLYIVVEDDGPGIPLSKREVIFDRGQRVDTLRPGQGVGLAVAREITEQYEGKIVAGESMLGGARMEVIFGRQHSAPKDE

> PmrD MEWLVKKSCCNKQDNRHVLMLCDAGGAIKMIAEVKSDFAVKVGDLLSPLQNALYCINREKLHTVKVLSASSYSPDEWERQC**T**AAGKTQ

> MgrB

MKKFRWVVLVVVVLACLLLWAQVFNMMCDQDVQFFSGICAINQFIPW

Binsker U, Käsbohrer A, Hammerl JA. Global colistin use: a review of the emergence of resistant Enterobacterales and the impact on their genetic basis. FEMS Microbiol Rev. 2022 ;46(1):fuab049. doi: 10.1093/femsre/fuab049.
