# Supplementary material for: First detection and characterization of mcr-1 colistin resistant E. coli from wild rat in Bangladesh
Source: PLoS One. 2024 May 14;19(5):e0296109. doi: 10.1371/journal.pone.0296109 (PMC11093362; doi:10.1371/journal.pone.0296109)
Supplement: Attachment — Submitted filename: Response to Reviewers-PONE-D-23-39379-2ndrev-28032024.docx [file pone.0296109.s010.docx]

**Response to Reviewers**

The authors sincerely appreciate the reviewer for providing insightful perspectives and valuable suggestions on the manuscript. We have carefully addressed all queries and incorporated the suggested modifications. Below, we present a point-by-point response to the reviewer's comments for your thoughtful consideration:

Reviewer #2: This manuscript has been revised on first round. I have some comments to the authors below:
1. Why the author detect only mcr-1 to mcr-8? Why not mcr-9 and mcr-10?

**Response:** Thank you for your nice comment. The prevalence of mcr-1 is extensive, whereas occurrences of mcr-2 to mcr-10 have been reported sporadically. Given the laboratory resources available during the experiment, we initially investigated the presence of mcr-1 to mcr-8. However, in response to the reviewer's query and to prevent any confusion, we conducted additional analysis on the samples for mcr-9 and mcr-10. Unfortunately, none of the isolates tested positive for the mcr-9 or mcr-10 genes. We have diligently updated this information in the manuscript to present a comprehensive overview (Ln. 96, 102-103, 146, 248-249 in the Track changed version, and Supplementary Table 1).

2. Although the author detected one E. coli isolate which carrying mcr-1 and colistin was also resistance, but it is not guarantee that this phenomenon of colistin resistance was due to mcr-1.
Chromosomal-mediated colistin resistant genes are also important. I recommend the author should extensive additional analysis of these chromosomal-gene mutations because of you have WGS data of mcr-1-harboring isolate. You can follow this paper as a guideline for these genes.
3. Please discuss your result of (2) in discussion part.

Binsker U, Käsbohrer A, Hammerl JA. Global colistin use: a review of the emergence of resistant Enterobacterales and the impact on their genetic basis. FEMS Microbiol Rev. 2022;46(1):fuab049. doi:10.1093/femsre/fuab049

**Response:** We extend our sincere gratitude to the reviewer for their valuable suggestion. Our investigation delved into the whole-genome sequencing (WGS) and subsequent analysis of chromosomal genes potentially implicated in the development of colistin resistance. Following the guidance provided in the manuscript mentioned by the reviewer, we meticulously scrutinized the aa sequences of PmrCAB, PmrD, PhoPQ, and MgrB.

Our analysis revealed no mutations in the aa sequences of PmrA, PmrC, PhoP, PhoQ, and MgrB. However, we did detect two mutations in PmrB at positions D283G and Y358N, along with one mutation in PmrD at position K82T. These mutations were previously identified in colistin-resistant strains of *E. coli*, albeit lacking experimental confirmation.

It's worth noting that we did not conduct experimental validation in this study, thus we cannot conclusively rule out the potential chromosomal contribution to the colistin resistance observed in our strain. We have elaborated on this matter in our manuscript (Ln. 250-260, Track changed version) and provided the gene sequences as supplementary data (Supplementary data 3) for your thoughtful consideration.

Reviewer #3: lines 90. 91 - Is MacConkey selective for E. coli?

**Response:** MacConkey agar is indeed selective for Gram-negative bacteria, particularly for members of the Enterobacteriaceae family, such as *Escherichia coli*. One reason why MacConkey agar is commonly used for isolating *E. coli* from crude samples like feces or food is because it not only selects for Gram-negative bacteria but also differentiates them based on their ability to ferment lactose. *E. coli*, being a lactose fermenter, produces colonies that appear pink to red on MacConkey agar. This selective and differential medium helps in the isolation and identification of *E. coli* from mixed microbial populations. Other than MacConkey, EMB agar (Eosin Methylene Blue agar) is another selective and differential medium commonly used for isolating Gram-negative bacteria, including *E. coli*. However, both media are suitable for isolating *E. coli*. MacConkey agar is commonly used in our laboratory and long been used in our lab for its simplicity and effectiveness in differentiating lactose fermenters, which is particularly useful when isolating *E. coli* from samples with potentially high microbial diversity, such as feces or certain food samples.

line 168-169 - A total of seven (7) plasmids belonging to IncHI2, IncHI2A, IncI2(delta), IncN, IncX1 169 and p0111 replicon families with > 98% identity were detected in the genome by PlasmidFinder. -Does the software detect plasmid replicons or plasmids?

**Response:** PlasmidFinder does not directly detect entire plasmids, it provides information about the presence of specific plasmid replicons within bacterial isolates, which can be indicative of the presence of certain types of plasmids. Replicon sequences from 559 fully sequenced plasmids associated with the family Enterobacteriaceae in the NCBI nucleotide database were collected to build a consensus database for integration into PlasmidFinder that can be used for replicon sequence analysis of raw, contig group, or completely assembled and closed plasmid sequencing data. The PlasmidFinder database currently consists of 116 replicon sequences that match with at least at 80% nucleotide identity all replicon sequences identified in the 559 fully sequenced plasmids.

158 - Antibiotic susceptibility through disc diffusion identified 58.82 % CREC as MDR (Table 2). - Table 2 shows antibiotic resistance patterns with no mention of MDR statistics.

**Response:** We appreciate your gracious feedback. We would like to draw your attention to Table 2, specifically the left panel, where we have highlighted the Number of antimicrobial classes. As described in the manuscript, specifically lines 115 to 116 (Track changed version), we defined isolates exhibiting resistance to three or more classes of antibiotics as MDR. We trust this clarification fulfills the necessary criteria.

Fig 1: Has bad resolution

**Response:** Figure 1 has been optimized to comply with the journal's specifications. Following your suggestion, we have further enhanced the resolution of Figure 1.

Fig 3: An overview of the antimicrobial resistance genes (ARGs) (A), resistant antibiotics (B), and resistance mechanisms (C), in E. coli RJWEcMCR-1-BAU. - What do you mean by resistant antibiotics?

**Response:** Apologies for any confusion caused by our previous heading. We've revised it accordingly to clarify that "resistant antibiotic" refers to antimicrobial resistance genes (ARGs) detected across various antimicrobial classes. Thank you for your understanding.

Fig 4: The plasmid sequence was derived by mapping the raw reads with E. coli plasmid pHLJ109-25 - This is not clear. Where the authors able to map a plasmid using illumina sequencing. Because of the sequencing depth it is generally impossible to map entire plasmids using the technique. Hence, there is reference to plasmid replicons as these can be predicted from the contigs. The authors seem to have been able to accurately identify two plasmids one carrying the mcr- 1 gene (The mcr-1 gene was located on a 60 kb IncI2 plasmid.) and another one carrying a myriad of ARGs (on a 288 kb mega-plasmid separately). Are the authors reporting similarity of their raw reads with plasmids or did they actually map plasmids as reported?

**Response:** We express our sincere gratitude to the reviewer for their insightful query. The advancements in technology over recent years have been remarkable, particularly in the realm of genomics. Mapping high-throughput data has become a swift process with modern genomic workbenches such as Biomatters (Geneious Prime) and CLC Genomics Workbenches.

In this study, we initially conducted a BLAT search to identify sequence similarity of the mcr-1.1 gene and other antimicrobial resistance-containing contigs. Plasmids exhibiting 100% similarity were then downloaded, and the raw reads were subsequently mapped against these downloaded plasmids using Geneious Prime version 2022.1.1, allowing us to generate the plasmid sequences referenced in our study. While we do not report similarity with the plasmids, we successfully recovered the plasmids as described in the manuscript.

For enhanced clarity, the plasmid sequences obtained through mapping have been submitted as supplementary files (S2 Data and S3 Data) in the current version of the manuscript.

There's need for clarity on how many E. coli isolates were obtained from the 39 rats. How many isolates from each rat. Clarity is also required on the the 17 CREC. The MIC results of all 17 should be presented. The mcr-1 positive isolate was clearly colistin resistant (MIC of ≥ 8 µg/ml) but the others were susceptible ≤ 2 µg/mL according to EUCAST guidelines. Why were all 17 isolates considered CREC?

**Response:** We apologize for the inadequate and ambiguous description provided. In our study, we processed fecal samples by suspending 3 to 4 pellets in 1 ml of PBS and directly inoculated them onto MacConkey agar plates supplemented with colistin sulfate, without prior enrichment. It's worth noting that not all fecal samples yielded growth.

A total of 40 *E. coli* colonies were recovered from the 39 rats examined and were confirmed using *E. coli*-specific *malB* gene targeted PCR. Subsequently, all colonies underwent screening for *mcr* genes and antimicrobial resistance through disc diffusion. Interestingly, colonies originating from the same rat exhibited identical patterns on *mcr* PCR and antimicrobial resistance, leading us to consider them as a single entity. Following these criteria, we identified 17 colistin-resistant *E. coli* (CREC) isolates from 17 positive rats.

Among these isolates, only those testing positive for *mcr-1* displayed a minimum inhibitory concentration (MIC) of ≥ 8 µg/ml, while all others exhibited MIC values of ≥ 2 µg/ml (i.e., < 4 µg/ml). However, we did not determine the MIC fraction between 2 and 4 µg/ml. Considering the breakpoints established by the Clinical and Laboratory Standards Institute (CLSI) and the European Committee on Antimicrobial Susceptibility Testing (EUCAST), all isolates were deemed resistant to colistin, thereby classifying the 17 isolates as CREC.

Furthermore, it's important to highlight that according to CLSI guidelines, polymyxin resistance is categorized as intermediate or resistant, with no susceptible category. It's crucial to note that the intermediate category does not imply sensitivity but rather resistance to the antimicrobial agent.
